# Linkage disequilibrium based genotype calling from low-coverage shotgun sequencing reads

**DOI:** 10.1186/1471-2105-12-S1-S53

**Published:** 2011-02-15

**Authors:** Jorge Duitama, Justin Kennedy, Sanjiv Dinakar, Yözen Hernández, Yufeng Wu, Ion I Măndoiu

**Affiliations:** 1 Department of Computer Science & Engineering, University of Connecticut,371 Fairfield Rd., Unit 2155, Storrs, CT 06269-2155, USA; 2 Department of Computer Science, University of Maryland,College Park, Maryland 20742, USA; 3 Department of Computer Science, Hunter College,695 Park Avenue, New York, NY 10021, USA

## Abstract

**Background:**

Recent technology advances have enabled sequencing of individual genomes, promising to revolutionize biomedical research. However, deep sequencing remains more expensive than microarrays for performing whole-genome SNP genotyping.

**Results:**

In this paper we introduce a new multi-locus statistical model and computationally efficient genotype calling algorithms that integrate shotgun sequencing data with linkage disequilibrium (LD) information extracted from reference population panels such as Hapmap or the 1000 genomes project. Experiments on publicly available 454, Illumina, and ABI SOLiD sequencing datasets suggest that integration of LD information results in genotype calling accuracy comparable to that of microarray platforms from sequencing data of low-coverage. A software package implementing our algorithm, released under the GNU General Public License, is available at http://dna.engr.uconn.edu/software/GeneSeq/.

**Conclusions:**

Integration of LD information leads to significant improvements in genotype calling accuracy compared to prior LD-oblivious methods, rendering low-coverage sequencing as a viable alternative to microarrays for conducting large-scale genome-wide association studies.

## Background

Recent advances in massively parallel sequencing have dramatically increased throughput compared to the classic Sanger technology, with several commercially available platforms including 454, Illumina, ABI SOLiD, and Helicos delivering billions of bases per day. This has enabled sequencing of several individual genomes [1-8], ushering the era of personal genomics. Thousands of other individual genomes are currently being sequenced as part of large scale projects such as the international 1000 genomes project [9], and whole genome sequencing is likely to become routine as sequencing costs continue to decrease. However, analysis of whole genome sequencing data remains challenging [10] and experimental design optimization has only recently started to receive attention [11].

In this paper we focus on one of the most fundamental genomic analyses, namely determining the genotypes at known loci of genome variation such as single nucleotide polymorphisms (SNPs). Diploid organisms including humans inherit two (possibly identical) variants or *alleles* at autosomal loci, and most medical applications of personal genomics require accurate identification of both variants, the combination of which is referred to as *genotype*. Of particular interest are loci that are heterozygous, i.e., loci for which the two chromosomes carry different alleles. However, identifying heterozygous loci from low-coverage whole-genome sequencing data poses a significant challenge. Sequencing data is obtained using the so called “shotgun” approach, whereby millions of short DNA fragments called reads are generated from randomly selected locations on the two chromosomes. If, for example, there are only two reads generated from a heterozygous locus, there is a 50% chance that one allele would be missed. To compensate for sequencing errors, existing methods for detecting heterozygous loci have even higher minimum allele coverage requirements, e.g., in [3,8], calling an allele requires the presence of at least two reads supporting it. Consequently, due to the relatively low sequencing depth used in these two studies (about 7.5×), the reported sensitivity of detecting heterozygous SNPs was of only 75%.

A simple way to improve genotype calling accuracy is to increase sequencing depth, as the probability of “missing” an allele decreases with the number of reads. After taking into account the effect of sequencing errors it has been estimated that, in the absence of additional information, achieving 99% sensitivity at detecting heterozygous SNPs would require an average sequencing depth of over 21× [12]. Our main contribution is to demonstrate that high accuracy SNP genotypes can be inferred from shotgun sequencing data of much lower depth by exploiting the correlation between alleles at nearby SNP sites, commonly referred to as *linkage disequilibrium* (LD).

LD patterns over millions of common SNPs have been mapped for several populations as part of the Hapmap project [13]. The strong LD observed in human populations has already been exploited by methods for imputation of genotypes at untyped SNP loci based on nearby SNP genotypes [14-19], see [20] for a recent review, and more recently, for improving genotype calling accuracy from microarray hybridization signals [21]. Another striking demonstration of the power of LD has been the inference of Watson’s APOE status [22] despite the removal of sequencing reads covering this region from the published dataset [8]. In this work we introduce a novel hierarchical factorial Hidden Markov Model (HMM) that allows integrated analysis of LD information extracted from reference population panels such as Hapmap and short-read sequencing data generated by current technologies. Although the ensuing multilocus genotype inference is computationally hard, we develop a scalable heuristic similar to the posterior decoding algorithm for HMMs. A software package implementing this algorithm has been released under the GNU General Public License and is available at http://dna.engr.uconn.edu/software/GeneSeq/. We also present experimental results on publicly available 454, Illumina, and ABI SOLiD whole-genome sequencing datasets showing that integration of LD information leads to significant improvements in genotype calling accuracy compared to prior LD-oblivious methods. For example, at 6× average mapped read coverage, our algorithm calls heterozygous SNP genotypes with about 96% accuracy, and accuracy can be further increased to 98-99% by leaving uncalled a small percentage of SNP genotypes with low posterior probabilities. This accuracy is comparable to that achieved by microarray-based genotyping platforms. Coupled with continued decreases in sequencing costs, the reduced sequencing depth required when using LD information renders low-coverage sequencing as a potentially more cost-effective alternative to microarrays for the next generation of genome wide association studies (GWAS). For example, the ABI SOLiD 4hq is expected to deliver 300Gb of sequencing data per run, or the equivalent of 16 individual genomes at 6× coverage, with a cost of only $600 per genome [23]. Undoubtedly, cost will be an important factor in future GWAS studies, which are expected to use much higher sample sizes compared to past studies in order to enable the study of gene-gene and gene-environment interactions [24].

## Methods

In this section we begin by describing a simplified statistical model that assumes independence between loci, then extend it to include dependences between alleles at different SNPs due to LD. We next formalize the multilocus genotype calling problem in the context of the extended model and show that computing the most likely multilocus genotype is computationally hard. Finally, we present a posterior decoding heuristic which independently selects the most likely genotype at each locus conditional on the entire set of reads.

### Notations

We use uppercase italic letters (e.g., *X*) to denote random variables and lowercase italic letters (e.g., *x*) to denote generic values taken by them. Vectors of random variables and generic values are denoted by boldface uppercase (e.g., **X**), respectively boldface lowercase letters (e.g., **x**). When there is no ambiguity on the underlying probabilistic event we use *P*(*x*) to denote *P*(*X* = *x*), with similar shorthands used for joint and conditional probabilities of multiple events. For simplicity we consider only bialelic SNPs on autosomes. For every SNP locus, we denote the two possible alleles by 0 and 1, and the three genotypes by 0,1, and 2, with 0 and 2 denoting the homozygous 0 and homozygous 1 genotypes, and 1 denoting the heterozygous genotype.

### Single SNP genotype calling

In this section we describe a genotype inference model that assumes the SNPs to be unlinked as in [8], but further incorporates allele uncertainty quantified by sequencing quality scores, read mapping uncertainty, and population genotype frequencies estimated from a reference panel.

Let *r* be a read mapped onto the genome. If *r* covers SNP locus *i*, we denote by *r*(*i*) the allele observed in the read at this locus. Since our focus is on genotyping SNPs represented in a reference panel, we further assume that panel SNPs at which the individual under study has novel allele variants (observed in [8] at only 0.02% of the markers) have been identified in a preliminary analysis, e.g., by using binomial probability test of [8]. Based on this assumption, all reads with alleles not represented in the panel population are ignored, and for remaining reads *r* we have that *r*(*i*) ∈ {0,1}. The probability that allele *r*(*i*) is affected by a sequencing error is denoted by *ε_r_*_(*i*)_. In our experiments we set *ε_r_*_(*i*)_ = 10^*-q_r_*_(*i*)_/10^, where where *q_r_*_(_*_i_*_)_ denotes the Phred quality score of *r*(*i*) [*25*].

Let *G_i_* be a random variable denoting the unknown SNP genotype at locus *i*, and let **r***_i_* = {*r_i_*,_1_, … ,*r_i,c_i__*} be the arbitrarily ordered set of shotgun reads covering locus *i*, where *c_i_* is the coverage at this locus. Since for a homozygous genotype the allele of origin for a read is the same regardless of which chromosome is sampled, we get:(1)

and(2)

For a read *r* covering a heterozygous SNP locus *i* allele *r*(*i*) can be observed either as the result of sampling *r* from the chromosome bearing allele *r*(*i*) and correctly sequencing it, or as the result of sampling the other chromosome followed by a sequencing error. Hence:(3)

A natural approach to single-locus SNP genotyping is to call a genotype of *ĝ_i_* = argmax *_g_i__*_∈{0,1,2}_*P*(*g_i_*|**r***_i_*) for every SNP locus *i*, where the posterior probabilities *P*(*g_i_*|**r***_i_*) are obtained from (1)-(3) by applying Bayes’ formula:(4)

and *P*(*G_i_* = *g*) denotes the population frequency of genotype *g*, estimated from the reference panel. If read mapping uncertainty is available in the form of probabilities *m*(*r*) that read *r* is mapped at the correct position, such information can be accounted for in genotype calling by replacing the above conditional probabilities with genotype weights obtained from (1)-(3) by rising the terms corresponding to read *r* to power *m*(*r*). Although the resulting weights can no longer be interpreted as conditional genotype probabilities, they naturally allow interpreting the presence of a read *r* with mapping confidence *m*(*r*) < 1 as the equivalent of observing an *m*(*r*) fraction of an identical read mapped with confidence 1.

### A statistical model for multilocus genotype inference

**Figure 1:**
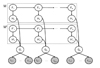
HF-HMM model for multilocus genotype inference.

In this section we introduce a statistical model that allows us to integrate shotgun sequencing data and LD information in the inference of SNP genotypes. Our model, represented graphically in Fig. 1, can be thought of as a *hierarchical factorial HMM* (HF-HMM). Indeed, we use a distributed state (characteristic of factorial HMMs [26]) to exploit the independence between maternal and paternal chromosomes (implied by the assumption of random mating), while also employing a multilevel state representation as in hierarchical HMMs [27] to capture the structured nature of the data. This structure leads to a reduced number of model parameters and enables highly scalable inference algorithms.

At the core of the model are two left-to-right HMMs *M* and *M′* (dotted boxes in Fig. 1), each emitting haplotypes with frequencies corresponding to those in the populations of origin for the sequenced individual's parents. Under *M* and *M′,* each haplotype is viewed as a mosaic formed as a result of historical recombination among a set of *K* founder haplotypes, where *K* is a population specific model parameter. Formally, for every SNP locus *i* ∈ {1, … , *n*}, we let  be a random variable representing the allele observed at this locus on the maternal (paternal) chromosome of the individual under study, and  be a random variable denoting the founder haplotype from which *H_i_* (respectively ) originates. As in previous works [15,17,28-30], we assume that *F_i_* form the states of a first order HMM with emissions *H_i_*, and estimate probabilities *P*(*f*_1_), *P*(*f_i_*_+1_|*f_i_*), and *P*(*h_i_*|*f_i_*)) using the classical Baum-Welch algorithm [31] based on haplotypes inferred from a panel representing the population of origin of the individual’s mother. Probabilities , and  are estimated in the same way based on haplotypes inferred from a panel representing the population of origin of the individual’s father.

We define  to be 1 if  and 0 otherwise. Finally, assuming that each read covers no more than a SNP locus, we set(5)

This implies that *P*(**r***_i_*|*g_i_*) are given by equations (1)-(3), and in the following we will assume that probabilities *P*(**r***_i_*|*g_i_*) are precomputed in *O*(*m*) time, where  is bounded above by the total number of reads. We can now formulate the following:

### Multilocus Genotyping Problem (MGP)

**Given:***Trained HMM models M, M′ and set of shotgun reads***r** = (**r**_1_, … , **r***_n_*)

**Find:***Multilocus genotype*g* ∈ {0,1,2}*^n^ with maximum posterior probability, i.e.,*

g* = argmax_**g**_*P*(**g**|**r**, *M*, *M′*)        (6)

### Computational complexity

In this section we show that MGP is NP-hard. Let *Maximum Multilocus Genotype Probability Problem (MMGPP)* denote the optimization version of MGP that requires finding max_**g**_*P*(**g**|**r**, *M*, *M′*).

**Theorem 1**. *For any ∊ >* 0, *MMGPP cannot be approximated within**unless P=NP, and it cannot be approximated within**unless ZPP=NP. Furthermore, this holds even if M′ = M*.

**Figure 2:**
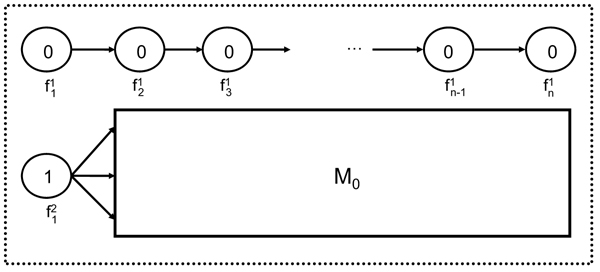
Schematic state diagram for the HMMs *M* and *M*′ used in the reduction of the consensus string problem to MMGPP.

*Proof*. Lyngsø et al. [32] give an approximation preserving reduction from the clique problem to the problem of computing the maximum probability of a string emitted by an HMM. It is not difficult to modify their construction to show that this reduction holds for left-to-right HMMs that emit 0/1 strings of fixed length. Next, we show that computing the maximum probability of a string emitted by such an HMM *M*_0_ can be reduced in approximation preserving manner to MMGPP with *M′* = *M*. The haplotype models *M* and *M′* are obtained from *M*_0_ as follows (see the schematic state diagram in Fig. 2):

• The number of SNPs *n* is set to one plus the length of the strings emitted by *M*_0_.

• At the first SNP, for two founder states  and  we have ; all other founder states have zero initial probability.

• For every SNP locus *i* > 1 we add a new founder  as well as a set of founders corresponding to the states at “column” *i* — 1 of *M*_0_.

• All founder , emit 0 with probability 1. Furthermore,  for every *i* = 2, … , *n*.

• Founder  emits 1 with probability 1, and has transitions to founders , according to the initial probabilities of *M*_0_.

• All other emission and transition probabilities are identical to those for the corresponding states of *M*_0_.

Finally, we set **r** = {*r*_0_, *r*_1_} where *r*_0_ is a read that supports allele 0 at first SNP and *r*_1_ is a read that supports the allele 1 at first SNP. Error probabilities for both alleles are set to zero.

Note that *P*(**g**|**r**, *M*, *M′*) ≠ 0 only for multilocus genotypes with *g*_1_ = 1 and *g_i_* ∈ {0,1} for *i* = 2, … , *n*.

Furthermore, for such a genotype **g**,(7)

The last equality comes from the fact that **g** can only be observed when the maternal haplotype is 0*^n^* and the paternal haplotype is **g** or vice-versa, and each of these configurations have a probability of *P*(*g*_2_, … , *g_n_*|*M*_0_)/4. The innaproximability result follows from [32] since, by (7), *P*(**g**|**r**, *M, M′*) is constant fraction of *P*(*g*_2_, … , *g_n_*|*M*_0_).

Since an algorithm similar to the forward algorithm for HMMs can be used to compute in polynomial time the marginal probability of a given genotype, Theorem 1 implies the following:

**Corollary 2.***MGP is NP-Hard.*

### Posterior decoding algorithm

We next present an MGP heuristic similar to the posterior decoding algorithm for HMMs. Specifically, the algorithm selects for each SNP locus *i* the genotype *ĝ_i_* with maximum posterior probability given the read data **r**. Note that, unlike the single SNP genotype calling method, where we condition only on the set **r***_i_* of reads overlapping locus *i*, in the posterior decoding algorithm we take into account the *entire* set of reads:

Posterior decoding algorithm

*Step 1. For each i* = 1, … , *n, ĝ_i_* ← argmax*_g_i__P*(*g_i_*|**r**)

*Step 2. Return***ĝ** = (*ĝ*_1_, … , *ĝ_n_*)

Below we detail an *O*(*m + nK*^3^) implementation of the posterior decoding algorithm. Since *P*(*g_i_*|**r**) ∝ *P*(*g_i_*, **r**), for implementing the maximization in Step 1 it suffices to compute marginal probabilities *P*(*g_i_*, **r**) for every *i* = 1, … , *n* and *g_i_* ∈ {0,1,2}. For each SNP locus *i* and each pair of founders  we let the *forward probability* be  and the *backward probability* be  respectively. Using these forward and the backward probabilities, the marginal probability *P*(*g_i_*, **r**) can be written as

where  is given by:

Thus all probabilities *P*(*g_i_*, **r**) can be computed in *O*(*nK*^2^) once the forward and backward probabilities  and  are available.

The forward probabilities can be computed using the recurrence:(8)(9)

for every  and *i* = 2, … , *n*, where(10)

The inner sum in equation (9) is independent of *f*_i_, and so its repeated computation can be avoided by replacing (9) with:(11)(12)

A similar optimization can be applied when computing the backward probabilities, resulting in the following recurrence:(13)(14)(15)

Forward and backward probabilities can thus be computed in *O*(*nK*^3^) by using recurrences (8), (11), and (12), respectively (13), (14), and (15), resulting in an overall runtime of *O*(*m + nK*^3^), where *m* is the number of reads, *n* is the number of SNPs, and *K* is a user selected parameter denoting the number of founders in the HMM models of haplotype diversity in the parental populations (we used *K* = 7 in our experiments).

## Results and discussion

### Datasets

We evaluated the HMM-based posterior decoding algorithm on shotgun sequencing datasets generated using three different sequencing technologies, as follows:

1. Watson 454: A set of 74.4 million reads downloaded from the NCBI SRA database (submission number: SRA000065). The reads, with an average length of ~265 bp, were generated using the Roche 454 FLX platform as part of James Watson’s personal genome project. This is a subset of the 106.5 million 454 reads analyzed in [8]. Unless noted otherwise, the haplotype panel used to train identical HMM models for the maternal and paternal populations was obtained by phasing CEU trio genotypes from Hapmap r23a [13] using the ENT algorithm of [33] and retaining parent haplotypes from each trio. As in [8], genotype calling accuracy was assessed using the SNP genotypes determined using duplicate hybridization experiments with Affymetrix 500k microarrays (only concordant genotypes were retained in the test set).

2. NA18507 Illumina: A set of 525 million paired-end reads downloaded from the NCBI SRA database (submission number: SRA000271). These 36bp reads, which were generated using the Illumina Genome Analyzer from a Hapmap Yoruban individual identified as NA18507, are a subset of the dataset analyzed in [1]. For the analysis of this dataset the HMM models for maternal and paternal populations were trained using YRI haplotypes from Hapmap r22, excluding the haplotypes of the YRI trio that contains NA18507. As gold standard we used the genotypes published as part of Hapmap r22 for individual NA18507.

3. NA18507 SOLiD: A set of 900 million single ABI SOLiD reads generated from Hapmap individual NA18507 was kindly provided by the authors of [4]. Reads varied in length between 20 and 44 bp, and were already mapped to the reference genome. Corresponding raw reads are available for download from the NCBI SRA database (submission number: SRA000272). HMM models and gold standard genotypes were determined in the same way as for the NA18507 Illumina dataset.

### Read mapping

We mapped 454 reads on build 36.3 of the reference human genome using the NUCMER tool of the MUMmer package [34] with default parameters. We discarded alignments matching less than 90% of the reference or with 10 or more errors (mismatches or indels). We then discarded surviving reads with multiple matching positions. We mapped the Illumina reads using MAQ version 0.68 [35] with default parameters. We discarded alignments with mapping probability less than 0.9 or with sum of quality scores of mismatching bases higher than 60 (filtering was performed using the “submap” command of MAQ). SOLiD reads were mapped using the SOLiD System Analysis Pipeline Tool (Corona Lite) as described in [4]. Table 1 shows for each dataset the numbers of test SNPs, initial and mapped reads, and the average coverage per SNP after mapping.

**Table 1:** Summary statistics for the three datasets used in evaluation

Dataset	Test SNPs	Raw Reads	Raw Sequence	Mapped Reads	Avg. Mapped SNP coverage
Watson 454	443*K*	74.2*M*	19.7*Gb*	49.8*M* (67%)	5.85×
NA18507 Illumina	2.85*M*	525*M*	18.9*Gb*	397*M* (78%)	6.10×
NA18507 SOLiD	2.85*M*	2.45*G*	75*Gb*	900*M* (37%)	9.85×

### Genotyping accuracy

To evaluate the effects of read coverage on genotype calling for each dataset of *m* mapped reads we created four subsets of sizes *m*/16, *m*/8, *m*/4 and *m*/2 by picking reads at random. For each subset we called genotypes using the HMM-based posterior decoding algorithm, the binomial test of [8] (with a threshold of 0.01), and the single SNP posterior probability described under Methods. We also included in the comparison genotype calls obtained by SOAPsnp [36] and MAQ [35], two widely used LD-oblivious Bayesian methods implemented in the SAMtools package [37]. Unfortunately we could not compare our method with similar tools developed as part of the 1000 genomes project [38,39], which have only become publicly available when this article was in press. We measured the accuracy of each genotype calling method by computing the percentage of SNP genotype calls that match the gold standard available for each dataset. As in previous papers [1,3,4,8], we separately report accuracy for homozygous and heterozygous SNPs.

**Figure 3:**
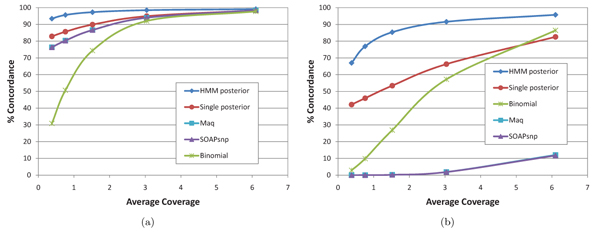
Genotype calling accuracy of compared methods for homozygous (a) and heterozygous (b) SNPs of the NA18507 Illumina dataset.

Fig. 3 shows genotype calling accuracy of the compared methods for varying average mapped read coverage on the NA18507 Illumina dataset; similar results were obtained on the other two datasets. For both homozygous and heterozygous SNPs, the posterior decoding algorithm has the highest accuracy of the compared methods at every considered coverage. The improvement in accuracy is most pronounced for heterozygous SNPs and at low average coverage. This is not surprising since, as previously noted in [3,4,8], at low average coverage there is an increasingly high probability of leaving uncovered at least one of the alleles of a heterozygous SNP, and a minimum coverage of each called allele is required by the binomial test, SOAPsnp, and MAQ. For example, the binomial test used in [3,8] requires that each allele be covered at least twice; in all our results we used the more relaxed requirement of covering each allele at least once. In contrast, the single-SNP posterior and the HMM-based posterior decoding algorithm do not have a minimum coverage requirement. By leveraging population allele frequencies estimated from the reference panel, the single-SNP posterior method already outperforms the binomial test, SOAPsnp, and MAQ at low average coverage. The HMM posterior decoding algorithm further improves accuracy by capturing LD information between neighboring SNPs.

**Figure 4:**
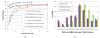
HMM posterior decoding accuracy (a) and distribution of reference allele coverage ratios for heterozygous SNPs (b) on the Watson 454, NA18507 Illumina, and NA18507 SOLiD datasets.

**Figure 5:**
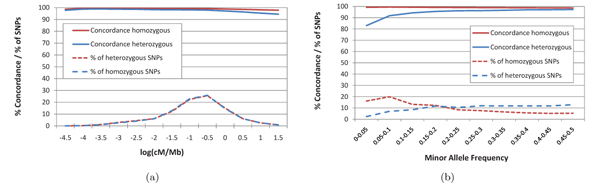
Effect of local recombination rate (a) and minor allele frequency (b) on concordance of genotypes called by the HMM posterior decoding algorithm on the NA18507 Illumina dataset.

Fig. 4(a) shows the accuracy achieved by the HMM posterior decoding algorithm when varying the average mapped read coverage for all three datasets. Genotyping accuracy achieved on the NA18507 Illumina reads matches that observed on Watson 454 reads for homozygous SNPs, and is only slightly lower for heterozygous SNPs. The accuracy achieved on the NA18507 SOLiD reads is consistently lower than that achieved for the other two datasets over the tested range of average coverages. We found that this difference is due to a bias towards the reference allele during color-to-base translation for reads mapped with Corona Lite. This bias is likely to induce incorrect heterozygous calls for some homozygous non-reference SNPs and homozygous reference calls for some heterozygous SNPs. The presence of this bias can be observed in Fig. 4(b), which shows the distribution of reference allele coverage ratios (i.e., ratios between the number of reference allele calls and the total number of mapped reads covering a locus) for heterozygous SNPs in the Watson 454, NA18507 Illumina, and NA18507 SOLiD datasets. In the absence of allele call biases, the average of reference allele coverage ratios over heterozygous SNPs should be close to 50%. We found that this was indeed the case for both the Watson 454 and NA18507 Illumina datasets (with averages of 51.39% and 51.02%, respectively) but not for the NA18507 SOLiD dataset (for which the average ratio is 63.02%). Fig. 5 shows the concordance of genotypes called by HMM posterior decoding on the NA18507 Illumina dataset for groups of SNPs with varying rates of local recombination, respectively minor allele frequency, both estimated from the YRI panel of Hapmap. The percentage of SNPs in each group is also plotted using dashed lines. For both homozygous and heterozygous SNPs concordance is relatively stable over the entire range of local recombination rates (see Fig. 5(a)), dropping below 96% only for heterozygous SNPs in regions with local recombination rate of over 10 cM/Mb. The effect of minor allele frequency is more pronounced (see Fig. 5(b)), with heterozygous SNPs concordance dropping to 83% for SNPs with minor allele frequency below 0.05. However, the overall accuracy is not affected too much since only 2% of heterozygous SNPs of NA18507 have an estimated allele frequency in this range.

**Figure 6:**
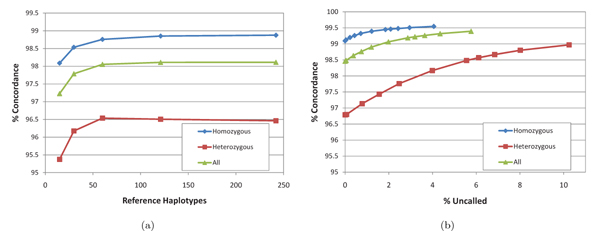
Effect of the reference panel size (a) and tradeoff between concordance and calling rate (b) for genotypes called by the HMM posterior decoding algorithm on the Watson 454 dataset.

To assess the effect of the size of the reference panel on genotyping accuracy, we conducted additional experiments on the Watson 454 reads using *N* = 242 CEU haplotypes available in Hapmap3. Similar to experiments with varying read coverage, we generated subsets of approximately *N*/16, *N*/8, *N*/4, and *N*/2 randomly selected reference haplotypes, and compared the accuracy achieved by running the HMM posterior algorithm using these subsets to that obtained using all *N* reference haplotypes. Fig. 6(a) gives the genotype call concordance obtained for different panel sizes. The results suggest that no significant improvement is achieved by increasing the reference panel size beyond 60-90. Thus – in contrast to methods for imputing untyped SNPs, which continue to benefit from increasing the panel size to several hundreds of haplotypes [40] – highly accurate genotype calling from sequencing data is possible with relatively small reference panels.

Since our algorithm computes a posterior probability for each SNP genotype, further increases in calling accuracy can be obtained at the expense of leaving uncalled a small percentage of SNP genotypes with low posterior probability. Such “no-calls” are commonly used in microarray-based genotyping for SNPs for which hybridization signals are ambiguous. Fig. 6(b) shows the tradeoffs achievable between the concordance and call rate when running the HMM posterior decoding algorithm on the full set of Watson 454 reads. Over all SNPs, concordance with the duplicate Affymetrix genotypes reaches 99.4% at a no-call rate of only 6%.

## Conclusions

In this paper we introduced a statistical model for multi-locus genotyping that integrates shotgun sequencing data with LD information extracted from a reference panel. Although finding the multi-locus genotype with maximum posterior probability under the integrated model is NP-Hard, experimental results suggest that a simple posterior decoding algorithm produces highly accurate genotype calls even from low-coverage sequencing data. Compared to current LD-oblivious genotype calling methods, our method allows researchers to achieve a desired accuracy target with reduced sequencing costs. For example, genotype calling accuracy achieved at 5-6× average coverage by a previously proposed binomial test is matched by the HMM-based posterior decoding algorithm using less than 1/4 of the reads. While a full comparison of sequencing and microarray based genotyping in the context of GWAS is beyond the scope of this paper, experimental results on three publicly available datasets generated using the 454, Illumina, and ABI SOLiD sequencing platforms suggest that at a mapped coverage depth of 5-6× our algorithm achieves an accuracy that is comparable to that of microarray platforms. Concordance rates reported for microarrays often exceed 99.9% (see, e.g., [41]), and are even higher for methods that integrate hybridization signals with LD information [21]. However, due to cost constraints, microarrays typically assay only a fraction of the SNPs represented in reference panels. For example, the next generation of Illumina microarrays is expected to assay only 5 million of the estimated 35 million SNPs generated by the 1000 genomes project [42]. Genotypes for the untyped SNPs would have to be inferred based solely on LD information, and even the best imputation methods have error rates of 5-6% [20], or 2-3% when leaving 10% of SNPs uncalled. Since the majority of SNPs must be imputed, this results in an overall accuracy below that achieved by the HMM posterior algorithm on the Watson 454 dataset.

In ongoing work we are exploring efficient algorithms for LD-based haplotype reconstruction from paired shotgun sequencing reads. We also plan to empirically compare our method with similar tools developed as part of the 1000 genomes project [38,39].

## Authors contributions

IIM and YW conceived the study. JD, JK, SD, and YH implemented the methods and conducted the experiments. JD and IIM drafted the manuscript. All authors participated in the development of the methods, data analysis, and manuscript revision. All authors have read and approved the final manuscript.

## Competing interests

The authors declare that they have no competing interests.

## References

[B1] Bentley D Accurate Whole Human Genome Sequencing using Reversible Terminator Chemistry Nature 2008 456 53 59 18987734 10.1038/nature07517 PMC2581791

[B2] Drmanac R Human Genome Sequencing Using Unchained Base Reads on Self-Assembling DNA Nanoarrays Science 2009 327 78 78 81 19892942 10.1126/science.1181498

[B3] Levy S The Diploid Genome Sequence of an Individual Human PLoS Biology 2007 5 10 e254+ 17803354 10.1371/journal.pbio.0050254 PMC1964779

[B4] McKernan K Sequence and structural variation in a human genome uncovered by short-read, massively parallel ligation sequencing using two-base encoding Genome Research 2009 19 1527 1541 19546169 10.1101/gr.091868.109 PMC2752135

[B5] Pushkarev D Neff N Quake S Single-molecule sequencing of an individual human genome Nature Biotechnology 2009 27 9 847 850 10.1038/nbt.1561 19668243 PMC4117198

[B6] Schuster S Complete Khoisan and Bantu genomes from southern Africa Nature 2010 463 18 943 947 10.1038/nature08795 20164927 PMC3890430

[B7] Wang J The diploid genome sequence of an Asian individual Nature 2008 456 60 65 18987735 10.1038/nature07484 PMC2716080

[B8] Wheeler D The complete genome of an individual by massively parallel DNA sequencing Nature 2008 452 872 876 10.1038/nature06884 18421352

[B9] The 1000 Genomes Project Consortium The 1000 Genomes Project Consortium http://www.1000genomes.org/

[B10] Snyder M Du J Gerstein M Personal genome sequencing: current approaches and challenges Genes & Development 2010 24 423 431 20194435 10.1101/gad.1864110 PMC2827837

[B11] Bashir A Bansal V Bafna V Designing deep sequencing experiments: detecting structural variation and estimating transcript abundance BMC Genomics 2010 11 385 10.1186/1471-2164-11-385 20565853 PMC3091630

[B12] Wendl M Wilson R Aspects of coverage in medical DNA sequencing BMC Bioinformatics 2008 9 239 18485222 10.1186/1471-2105-9-239 PMC2430974

[B13] The International HapMap Consortium A second generation human haplotype map of over 3.1 million SNPs Nature 2007 449 851 861 17943122 10.1038/nature06258 PMC2689609

[B14] Howie BN Donnelly P Marchini J A Flexible and Accurate Genotype Imputation Method for the Next Generation of Genome-Wide Association Studies PLoS Genet 2009 5 6 e1000529 19543373 10.1371/journal.pgen.1000529 PMC2689936

[B15] Kennedy J Măndoiu I Paşaniuc B Genotype Error Detection and Imputation using Hidden Markov Models of Haplotype Diversity Journal of Computational Biology 2008 15 9 1155 1171 10.1089/cmb.2007.0133 18973433

[B16] Li Y Abecasis GR Mach 1.0: Rapid Haplotype Reconstruction and Missing Genotype Inference American Journal of Human Genetics 2006 79 2290

[B17] Marchini J Howie B Myers S McVean G Donnelly P A new multipoint method for genome-wide association studies by imputation of genotypes Nature Genetics 2007 39 906 913 10.1038/ng2088 17572673

[B18] Stephens M Scheet P Accounting for decay of linkage disequilibrium in haplotype inference and missing-data imputation American Journal of Human Genetics 2005 76 449 462 15700229 10.1086/428594 PMC1196397

[B19] Wen X Nicolae DL Association studies for untyped markers with TUNA Bioinformatics 2008 24 435 437 10.1093/bioinformatics/btm603 18057020 PMC4051297

[B20] Marchini J Howie B Genotype imputation for genome-wide association studies Nature reviews. Genetics 2010 11 7 499 511 10.1038/nrg2796 20517342

[B21] Browning B Yu Z Simultaneous Genotype Calling and Haplotype Phasing Improves Genotype Accuracy and Reduces False-Positive Associations for Genome-wide Association Studies The American Journal of Human Genetics 2009 85 18 847 861 10.1016/j.ajhg.2009.11.004 PMC2790566 19931040

[B22] Nyholt DR Yu CE Visscher PM On Jim Watson’s APOE status: genetic information is hard to hide European Journal of Human Genetics 2008 17 2 147 149 18941475 10.1038/ejhg.2008.198 PMC2986051

[B23] Applied Biosystems SOLiD 4 System product description https://products.appliedbiosystems.com/

[B24] Burton PR Hansell AL Fortier I Manolio TA Khoury MJ Little J Elliott P Size matters: just how big is BIG?: Quantifying realistic sample size requirements for human genome epidemiology Int 2009 38 263 273 18676414 10.1093/ije/dyn147 PMC2639365

[B25] Ewing B Green P Base-calling of automated sequencer traces using phred. II. Error probabilities Genome Research 1998 8 3 186 194 9521922

[B26] Ghahramani Z Jordan M Factorial Hidden Markov Models Mach 1997 29 2-3 245 273 10.1023/A:1007425814087

[B27] Fine S Singer Y Tishby N The Hierarchical Hidden Markov Model: Analysis and Applications Mach 1998 32 41 62 10.1023/A:1007469218079

[B28] Kimmel G Shamir R A block-free hidden Markov model for genotypes and its application to disease association Journal of Computational Biology 2005 12 1243 1260 10.1089/cmb.2005.12.1243 16379532

[B29] Rastas P Koivisto M Mannila H Ukkonen E Phasing genotypes using a Hidden Markov model Bioinformatics Algorithms: Techniques and Applications, preliminary version Proc. WABI 2005 2008 Wiley 355 373

[B30] Schwartz R Algorithms for Association Study Design Using a Generalized Model of Haplotype Conservation Proc. CSB 2004 90 97 16448003

[B31] Baum L Petrie T Soules G Weiss N A maximization technique occurring in the statistical analysis of probabilistic functions of Markov chains Annals of Mathematical Statistics 1970 41 164 171 10.1214/aoms/1177697196

[B32] Lyngsø R Pedersen C The consensus string problem and the complexity of comparing hidden Markov models Journal of Computer Systems Science 2002 65 3 545 569 10.1016/S0022-0000(02)00009-0

[B33] Gusev A Mandoiu I Pasaniuc B Highly Scalable Genotype Phasing by Entropy Minimization IEEE/ACM Trans. on Computational Biology and Bioinformatics 2008 5 2 252 261 10.1109/TCBB.2007.70223 18451434

[B34] Kurtz S Versatile and open software for comparing large genomes Genome Biology 2004 5 2 R12 14759262 10.1186/gb-2004-5-2-r12 PMC395750

[B35] Li H Ruan J Durbin R Mapping short DNA sequencing reads and calling variants using mapping quality scores Genome Research 2008 18 1851 1858 18714091 10.1101/gr.078212.108 PMC2577856

[B36] Li R Li Y Fang X Yang H Wang J Kristiansen K Wang J SNP detection for massively parallel whole-genome resequencing Genome Research 2009 19 1124 1132 19420381 10.1101/gr.088013.108 PMC2694485

[B37] Li H Handsaker B Wysoker A Fennell T Ruan J The Sequence Alignment/Map format and SAMtools Bioinformatics 2009 25 16 2078 2079 19505943 10.1093/bioinformatics/btp352 PMC2723002

[B38] Li Y Abecasis G Thunder (beta version) 2010 http://genome.sph.umich.edu/wiki/Thunder

[B39] Le SQQ Durbin R SNP detection and genotyping from low-coverage sequencing data on multiple diploid samples Genome research 2010 10.1101/gr.113084.110 PMC3106328 20980557

[B40] Kennedy J Mandoiu I Pasaniuc B GEDI: Scalable Algorithms for Genotype Error Detection and Imputation Tech. Rep. 0911.1765, 2009 Cornell University arXiv e-print http://arxiv.org/abs/0911.1765

[B41] Hong H Su Z Ge W Shi L Perkins R Fang H Xu J Chen J Han T Kaput J Fuscoe J Tong W Assessing batch effects of genotype calling algorithm BRLMM for the Affymetrix GeneChip Human Mapping 500 K array set using 270 HapMap samples BMC Bioinformatics 2008 9 Suppl 9 S17 18793462 10.1186/1471-2105-9-S9-S17 PMC2537568

[B42] Illumina Empowering GWAS for a new era of discovery http://www.illumina.com/documents/products/technotes/technote_empower_gwas.pdf

